# Effect of burnout among physicians on observed adverse patient outcomes: a literature review

**DOI:** 10.1186/s12913-021-06371-x

**Published:** 2021-04-21

**Authors:** Kashan Yasin Mangory, Lavin Yadgar Ali, Karin Isaksson Rø, Reidar Tyssen

**Affiliations:** 1 Faculty of Medicine, University of Oslo, Oslo, Norway; 2 Institute for Studies of the Medical Profession, Oslo, Norway; 3 Department of Behavioural Medicine, Institute of Basic Medical Sciences, Faculty of Medicine, University of Oslo, Oslo, Norway

**Keywords:** Physician burnout, Patient outcome, Medical error

## Abstract

**Background:**

Physician burnout has potentially harmful effects for both physicians and their patients. Despite relationships between physician burnout and lowered patient satisfaction and clinician-rated adverse patient outcomes, there is scarce literature regarding effects on objective patient outcomes. This study aimed to examine the relationship between physician burnout and observed adverse patient outcomes via a review of the literature.

**Methods:**

A search was performed on the MEDLINE, EMBASE and PsychINFO databases, using keywords and Medical Subject Headings. The identified studies were in English, published from 2007 to 2019, measured burnout among physicians using the Maslach Burnout Inventory (MBI), and included observed adverse patient outcomes. In total, 360 eligible articles were identified, and 11 were included in the final review. All included studies measured patient outcomes by observed clinical measures (e.g. quality of care and medical errors).

**Results:**

Four studies found a clear significant relationship between physician burnout and observed adverse patient outcomes, while 6 did not. One study found a significant relationship with one of the MBI subscales. Burnout was, in contrast to depression, only partly associated with observed patient outcomes.

**Conclusions:**

This review illustrates the need for a validation of physician burnout measured by MBI with respect to observed patient outcomes. Further studies are required to investigate the effects of physician burnout on observed quality of their patient care.

## Background

Burnout among physicians is a widespread phenomenon and has been found to be of importance for both physicians and their patients [1]. Recent reviews suggest a link between physician burnout and negative impact on patient care, including increased medical errors [2–4]. A review study on psychosocial work stress and burnout from 2015 distinguished between different types of negative treatment outcomes, and described one category of more subjectively perceived outcomes, and two categories of objectively measured outcomes [5]: (I) The subjectively perceived outcomes were the physicians’ and/or patients’ perception of quality of care: These were outcomes related to patient centeredness of clinical activities and the physician–patient relationship as well as to physician empathy, accessibility, reliability and exchange of information. (II) One category of objectively measured outcomes included assessments of treatment success and absence of complications. These outcomes were evaluated by different observed indicators of treatment success (such as diabetes or blood pressure control), clinical quality or lack of treatment complications. (III) Another category of objectively measured outcomes was prevalence of medical errors: These outcomes included observed or recorded prescriptions, drug administration or surgical errors.

Most of the reviews so far build mainly on the subjectively perceived data, from physicians and patients themselves. There have been surprisingly few reviews of observational studies on the relationship between physician burnout and objectively measured outcomes of sub-optimal care of patients or errors [4]. We therefore need to study whether physician burnout is actually associated with observed adverse patient care and if it increases the risk of medical errors.

The distinction between objectively observed outcomes and subjective perception of quality of care is crucial for patients, physicians and their employers. We have therefore reviewed relevant observational studies to provide insights into this issue.

Burnout is usually measured using the Maslach Burnout Inventory (MBI). In a review of burnout prevalence, covering the period from 1991 to 2018, including 185 studies from 45 countries, 86% of the studies had used a version of the MBI [6]. The MBI has been found to have a replicable factor structure within and between professional groups and across countries [7]. Although there are several other valid measures of burnout (such as OLBI—Oldenburger Burnout Inventory, Burnout Inventory, CBI—Copenhagen Burnout Inventory), we have, on this background, limited the present review to include only studies using the MBI. The MBI has, however, been modified in several places in order to accommodate a variety of professionals and it has been shortened to include fewer items per dimension, or even only a single item [3].

The MBI includes three main components or subscales: emotional exhaustion (EE), which has been thought to be the initial reaction, followed by depersonalization/disengagement/cynicism (DP) which represents an emotionally detached attitude towards work (understood as a way of coping with exhaustion) and, finally, an experience of reduced personal accomplishment (or performance) (PA) [8]. Both cynicism and reduced accomplishment could indicate impaired physician functioning and subsequent subordinate patient care.

However, critique of this burnout model points to uncertainties about these possible relationships. Firstly, the MBI is not a diagnostic measure. Maslach surveyed persons “at work”, defining the third with highest score as “high burnout”. This has later been used as a cut-off score. Those who were off sick or not able to be at work were excluded from definition [9]. The scale is thereby poorly validated with respect to impairment and work performance [10, 11]. In an editorial from 2018 titled “Physician burnout—a serious symptom, but of what?” Schwenk and Gold discuss the high but varying prevalence of burnout reported in many studies and they strongly emphasize the need for a deeper understanding of its origins and possible consequences, which have not been sufficiently explained [12]. It is therefore important to relate burnout levels both to the physician’s individual suffering, but also to impact of burnout in the clinical setting and for patient treatment.

The relationships between job demands, burnout, work engagement, physician impairment and quality of patient care are complex. High job demands have been found to relate to emotional exhaustion, whereas lack of resources has been related to disengagement [13]. However, the doctor’s perception of the type of demand can influence how it impacts burnout. Demands perceived as challenges were found to be positively associated with work engagement, while demands perceived as hindrances could increase burnout. Engagement in work tasks could be developed despite a certain level of exhaustion [14], and could thus mitigate consequences of burnout. Additionally, there is a strong moral imperative in the profession to “put patients first”, articulated in the Declaration of Geneva’s Physicians´ Pledge [15], and this can urge doctors to preserve the quality of care even during heavy workload periods for the individual physicians.

In addition, a stressful working climate in some hospital units may lead to burnout also among health professionals other than physicians [16]. This applies also to primary health care settings in non-Western countries, as shown in recent studies [17, 18]. Burnout in whole hospital units and departments may also impact quality of patient care [5].

Several studies have shown that burned-out physicians themselves report that their condition affects their patient treatment and care negatively [2, 4]. A recent meta-analysis on the link between physician burnout and patient safety found that “physician burnout may jeopardize patient care”, but that most studies were cross-sectional and relied on outcomes self-reported by physicians [2]. The authors “failed to show significant links between physician burnout and patient safety outcomes recorded in the health-care systems (e.g., the health records of patients, monitoring etc.).” In line with this, another recent review found no significant link between physician burnout and observed clinical patient outcomes in any of the five studies identified; however, there were associations between physician burnout and outcomes reported by physicians and patients [4].

In this literature review, the definition of “adverse patient outcomes” includes observed measures for failure or lack of prevention, inadequate assessment (diagnoses) and treatment.

This literature review therefore aims to identify studies examining the association between physician burnout, measured by the MBI, and observed quality of care among their patients (including number of medical errors).

## Methods

We used the search engines MEDLINE, EMBASE, and PsychINFO. The final search consisted of keywords and Medical Subject Headings for predictor (e.g., burnout), sample (e.g., physician) and potential outcomes (e.g., medical errors) using the following three groups of search terms:

1. [Burnout, Professional/]OR [Physician burnout]
2. [Practice Patterns, Physicians/]OR [Physicians, Primary Care/]OR [Physicians, Family/]OR [Physicians/]OR [Primary Health Care/]OR [Internship and Residency/]
3. [Quality of Health Care/]OR [Medical Errors/]OR [Medication Errors/]OR [Physician-Patient Relations/]OR [Patient Care/]OR [Referral and Consultation/]OR [Attitude of Health Personnel/]OR [Patient Outcome]

The search combined “OR” between the search terms in each group with “AND” between the groups, resulting in 357 articles. In addition, experts in the field (KIR and RT) provided three articles [19–21]. The articles were subject to a manual review by two separate authors (KYM and LYA). Inclusion criteria were as follows: (I) the papers had abstracts written in English; (II) they were published between 2007 and 2019; (III) they measured physician burnout with MBI as a predictor; and (IV) they included observed measures of patient outcomes. The original search was for the student authors’ master thesis and had a 10-year frame (2007–2017). For the preparation of this manuscript, we updated the search to include the past 2 years, resulting in the range 2007–2019. The last year – 2020 – is heavily influenced by the COVID-19 pandemic at hospitals and other health services, and is not comparable to previous years with respect to possible confounders, such as, e.g., stress outside of work. First, we excluded articles that did not have an abstract or had an irrelevant title and abstract. The remaining 29 articles were then read in full text by the two first authors (KYM and LYA) and excluded if they did not include objective measures of patient outcomes or did not investigate the association between physician burnout, measured by the MBI, and adverse patient outcomes. The senior authors (KIR and RT) reviewed the articles and took actively part in the last selection and writing. Finally, 11 articles seemed to meet the inclusion criteria and were included in this review. Two of the studies included both nurses and physicians in intensive care units, which are considered stressful workplaces [16]. We included these studies because they comprised substantial samples of physicians.

## Results

Eleven studies were included in the review (Table 1). There was a large variation in the size of the samples, in the observed outcomes reported in the studies and in how MBI burnout was measured and defined (i.e. whether the subscales were dichotomized, categorized or used as continuous variables); see Table 1 footnotes.

**Table 1:** Description of studies retained by the literature review

Study	Country	Design	Sample	N	Response rate	Measured dimensions of burnout	Observed measure on quality of care	Association with burnout
Fahrenkopf et al. 2008 [22]	USA	Observational prospective cohort study	Paediatric residents	246	50%	EE and DP^a^	Missing or wrong prescription of drugs	Not associated with adverse patient treatment
Zantinge et al. 2009 [23]	Netherlands	Observation of video-recorded consultations and questionnaire	GPs	142	89%	EE, DP and PA^b^	Length of consultations, level of verbal communication, eye contact, and focus on psychosocial issues	PA associated with GPs communicating less affectively, being less patient-centred and less eye contact. EE and DP not associated with adverse patient treatment
Kushnir et al. 2014 [24]	Israel	Cross-sectional observational study	GPs/Primary care	136	99%	EE, DP and PA^c^	Number of referrals for diagnostic imaging, specialized health services or nurse sensitive treatments	Associated with more referrals
Yuguero Torres et al. 2015 [25]	Spain	Prospective observational study (1 year)	GPs	217	50%	EE, DP and PA^d^	Number of prescribed sick leaves	Not associated with prescribing more or longer sick leaves
Garrouste-Orgeas et al. 2015 [26]	France	Prospective observational study (2-year)	Doctors, nurses and care workers in intensive care unit	1988 (330 doctors)	77%	EE, DP and PA^e^	Medical errors (i.e., error of execution or error of planning). Adverse events were patient harms caused by medical interventions.	Not associated with adverse patient treatment
Welp et al. 2015 [19]	Switzerland	Observational study	Doctors and nurses in intensive care unit	1425 (243 doctors)	Not specified	EE, DP and PA^f^	Length of stay in hospital and standardized mortality ratio	Associated with increased standardized mortality ratio, but not length of stay
Pedersen et al. 2015 [27]	Denmark	Register study and questionnaire	GPs	835	72%	EE, DP and PA^g^	Number of requisitions for PSA among healthy male patients	Not associated with increased requisitions for PSA
Kwah et al. 2016 [28]	USA	Prospective observational study (1 year)	First-year residents in internal medicine	54	98% (initial), 59% (cohort)	EE and DP^h^	Medication prescription errors with potential for adverse drug effects	Not associated with increase in medical errors
Sun et al. 2017 [29]	USA	Cross-sectional observational study	Primary care	102	Not specified	EE, DP and PA^f^	Antibiotic prescriptions for acute respiratory infections	Not associated with increase in prescriptions
Noroxe et al. 2019 [20]	Denmark	Prospective observational study (6 months)	GPs	781	50.2%	EE, DP and PA^i^	Conditions not requiring hospitalization in the case of appropriate intervention in primary care (ambulatory care sensitive conditions)	Associated with increased frequency of hospitalizations of ambulatory care sensitive conditions
Noroxe et al. 2019 [21]	Denmark	Prospective observational study (6 months)	GPs	409	50.2%	EE, DP and PA^i^	Change of GP (unrelated to change of address)	Associated with increased likelihood of changing GPs

*Abbreviations:* *EE* emotional exhaustion, *DP* depersonalization, *PA* personal accomplishment (reduced), *GP* general practitioner, *PSA* prostate-specific antigen, *MBI* Maslach Burnout Inventory

^a^Defined burnout as EE > 27 *and* DP > 10

^b^EE, DP and PA were dichotomized into low and high scores, and outcomes reported for each dimension

^c^Overall burnout was measured as an average of the responses to items EE and DP (continuous variable)

^d^Scores for each category were divided into low, moderate and high

^e^Burnout measured as

(1) a combination of high EE and DP with low PA (dimensions dichotomized), and

(2) defined as over a cut-off of a global MBI score of 9

^f^Each dimension used as a continuous variable

^g^Burnout defined as high level of emotional exhaustion > 26

^h^Burnout defined as a high EE *or* high DP subscore (dichotomized variables)

^i^Burnout measured

1) in four quartiles for each dimension

2) As a *composite* score by adding up points corresponding to the quartile of each subscale (reversed score for personal accomplishment);

one point for scores in the first quartile, and two, three and four points for subsequent quartiles, respectively. The composite score was categorized into five groups, with increasing score indicating higher burnout

Seven studies were of general practitioners (GPs) or from primary care settings, two studies were from intensive care units, one study was among paediatric residents, and one study was about first-year residents in internal medicine. Three of the studies were from the United States, three from Denmark, and one study each from the Netherlands, Israel, Spain, France and Switzerland.

Fahrenkopf et al. [22] studied cohorts of paediatric residents from three U.S. hospitals over one and a half month. Burnout was measured by EE and DP in, and adverse patient outcome was the rate of errors made in ordering medication. Residents with high combined score for EE (> 27) and DP (> 10) were found to make similar rates of errors per resident month as residents with low burnout scores. There was a significant association between depression and medical errors (Harvard national depression screening day scale); depressed residents made 6.2 times more errors than non-depressed residents. The medical errors were collected and rated by trained observers (nurses and physicians).

Zantinge et al. [23] observed videotaped consultations in Dutch GPs. The findings were somewhat discrepant: GPs with low PA communicated less affectively, were less patient-centred and had less eye contact, whereas those with high EE and DP talked more about psychosocial problems in their consultations. The latter may have increased possibility for adequate mental health care.

Kushnir et al. [24] found in a study from Israel among primary care physicians that referral rates increased for both diagnostic tests and to specialist clinics among physicians who scored high on burnout. Being a specialist in family medicine was more important than burnout for referring to a specialist clinic. Burnout was here measured as a common mean of items of EE and DP.

Yuguero Torres et al. [25] studied doctors from 22 primary care centres in a region in Spain (Catalonia). The doctors’ prescribing of sick leave was not associated with higher burnout among them. Burnout scores were divided into low, moderate and high on EE, DP and PA. There was an association between higher burnout and lower empathy (Jefferson), but this did not influence the number or duration of sick leave prescriptions among the doctors.

Garrouste-Orgeas et al. [26] conducted a large and impressive 2-year prospective, observational study of doctors (*N* = 330), nurses and care workers in 31 Intensive Care Units (ICUs) in France. There was no significant association between medical errors or adverse events and burnout measured either as (1) a combination of high EE and DP with low PA, or (2) defined as over a cut-off on a global MBI score. There was a significant association between depression symptoms (CES-Depression Scale) and medical errors. Medical errors were defined by Delphi technique among 60 experts.

Welp et al. [19] studied patient safety in ICUs in 48 Swiss hospitals. They assessed standardized mortality ratios (SMR) and length of stay, and related these measures to burnout among medical doctors (*N* = 243) and nursing staff. The three burnout subscales were measured continuously, with EE predicting a significant increase in SMR, whereas DP and PA did not. Burnout did not predict length of stay for patients in ICU.

Pedersen et al. [27] studied the association between burnout and other psychological measures in Danish solo GP practices and testing of prostate specific antigen (PSA) in healthy male patients. Burnout measured as high EE (> 26) was not associated with increased PSA testing, and neither was empathy (Jefferson). On the other hand, high anxiety scores and bad outcome concerns among the GPs predicted increased incident PSA testing. PSA tests were obtained from register data.

Kwah et al. [28] studied first-year internal medicine residents in a smaller one-year prospective study in the United States. Surprisingly, there were fewer cumulative medical errors in residents with high burnout, measured as high EE or high DP scores. Other professionalism measures such as adequate time to complete discharge summaries and time to review laboratory tests results were not affected by burnout status.

Sun et al. [29] studied 36 primary care practices in Cleveland, Ohio, the United States. They found no association between either high burnout or low empathy (Jefferson) and antibiotic prescribing for acute respiratory infections. Burnout was measured continuously on all three subscales: EE, DP and PA. Prescription data were obtained from the patients’ medical records.

Noroxe et al. [20] conducted a large 6-month prospective study among Danish single-practice GPs and the number of hospitalisations of their patients. The number of hospitalisations for so-called ambulatory care sensitive conditions (that could potentially be managed by the GP) increased with increased level of burnout in the GP. All three burnout subscales, EE, DP and PA, were independent predictors of hospitalisation, and so was a composite burnout score of all subscales. The number of hospitalisations was obtained from national register data.

Noroxe et al. [21] did a second study of the same eligible sample of Danish single-practice GPs, as mentioned above. Here the outcome was change of GP among the patients (unrelated to their change of address). There was a dose-response like association between increase in DP and PA subscales (but not in EE) among the GPs and the likelihood that their patients left them for a new GP. There was also a significant association with an increase in the composite burnout score. Data regarding change of GP were obtained from a national register.

### Summary of the findings

Of the 11 studies, only 4 [19–21, 24] found a clear significant association between physician burnout and adverse patient outcomes, whereas 6 [22, 25–29] failed to find such an association. One study had divergent findings, with some aspects of patient care (observed communication skills) being reduced, whereas other aspects (time devoted to psychosocial issues) increased among burnout doctors [23]. There was no convincing association related to either positions or practice venue. Among the 7 studies from primary care/general practice, 3 found a clear significant association [20, 21, 24], while 4 did not [23, 25, 27, 29]. This discrepancy was the same with respect to the two ICU studies [19, 26] . Two studies (from Israel and Denmark) that investigated referral rates to specialist care and hospitalization from primary care/GPs found a significant association with physician burnout [20, 24]. Two studies that failed to show an association with burnout found an association between adverse events and measures of depression, which highlights that burnout and depression are different concepts with respect to impaired functioning [22, 26].

In studies where burnout has been measured by continuous variables (Kushnir et al., Welp et al.) [19, 24] or with variables divided into more than three categories (the two studies by Noroxe et al.) [20, 21], relationships to adverse care tended to be found. In the study by Sun et al. [29] no associations were found, despite a continuous burnout dimension measure. However, in this study, only 36 patients were included. Zantinge et al. [23] found discrepant results for the different dimensions of burnout measured above a cut-off. Associations between PA and observed poorer communication skills were found despite a dichotomous measure of burnout. However, this study was manually rated by observers, and hence more subject to individual assessments than were many of the other studies.

## Discussion

A major finding in our review was that physician burnout as measured by conventional use of MBI did not lead to observed adverse patient care in 6 of 11 studies. Four of the studies in the review found a clear link between burnout and observed adverse patient care, and one found a link between one of the burnout dimensions with respect to adverse patient outcomes, but not with the two other dimensions. Among the four studies with a clear association with adverse outcomes, two were regarding unnecessary referrals from GPs with burnout to specialist investigation or care, one was regarding the likelihood of the patient changing from a GP with burnout to a new GP, and one was about an outcome as serious as increased patient mortality when doctors and/or nurses reported burnout in an ICU.

Several other studies suggest that burnout is not a validated measure with respect to observed impaired functioning, unlike depression and other psychiatric conditions [10, 11]. In the original definition of burnout by Maslach, the levels of all three dimensions were required to be over a certain cut-off to denote burnout [30], but this does not necessarily associate with the cut-off levels of any clinical symptom that impairs functioning. In later studies, often only one or two dimensions have been used to define and measure burnout and some studies have used fewer or even just one item to define a dimension of burnout [31]. In our review, all four studies that were associated with adverse patient outcomes used the full three-dimension Maslach version, and they also utilized continuous variable data, or data divided into several (more than three) categories at the expense of dichotomization and categorization into few categories. By using all the variance in the variables, there is a greater chance of capturing any effects on the outcome.

More studies on this issue are thus needed. More recent Dutch and Swedish measures and assessments of an “emotional exhaustion syndrome” define validated cut-off levels that are used for classification of a disease or disorder that impairs functioning [11]. This can further the research on how burnout is related to patient care.

Some studies show a majority of participants simultaneously experience depressive symptoms and burnout [22, 32]. Although depressive symptoms and burnout share an appreciable amount of variance, the concepts are different entities [33]. However, burnout in more advanced stages has been found to lead to depressive symptoms [34]. Depressive symptoms can influence well-being and thereby work ability, with increased risk of mistakes or suboptimal functioning, which can, in turn, increase the risk of development of burnout [35]. The relationships between burnout, depressive symptoms and impaired functioning needs further study.

Many studies indicate that physicians with burnout believe they provide insufficient patient care [2, 4]. Physicians’ perceptions of their own abilities often do not correspond with observable evaluations of their work [36]. Self-criticism is common among students who choose to study medicine, and often reinforced by the studies and the work situation [37]. This makes self-reported data on own performance a less valid measure of observed performance. We know that some levels of self-criticism and even stress may drive performance, and the level of burnout among physicians that will result in observable adverse patient care remains to be identified [10].

Our review found more unnecessary referrals to specialist care among burned-out physicians, and one of the Danish studies found that the likelihood of patients changing their GP was higher in the case of GPs with burnout [21]. This may indicate that burnout affects aspects of the physician–patient relationship not captured by the measures of quality of patient care most commonly used presently [38], such as communication skills [39]. Zantinge et al.*’s* observation study of Dutch GPs and their patient-doctor relationship indicates this since the burnout doctors showed physical signs of more detached contact with their patients [23]. There is a need for further validation of the dependent measures of outcomes that are in use, to be able to understand this in a more nuanced way. On the other hand, one may argue that it is doubtful whether referral to specialist care or a patient choosing another primary care physician actually reduces observed quality of patient care. While it certainly increases costs of care and perhaps waiting time for the patients, a second opinion by another physician may be good for the patients with respect to quality of care. There may also be indirect effects on patient care from physician burnout, such as physicians’ sick leave or early retirement and, thereby, lack of continuity of care.

What about the six studies of physician burnout that showed no association with observed adverse patient outcome? A relevant theory suggests that patient care is maintained at the expense of the physicians’ well-being and mental health, which is known as the Conservation of Resources theory [40]. The profession might provide physicians with a work ethic that results in a high threshold of patient care, regardless of the favourability of their working conditions. It is known that burnout among physicians can lead to a corresponding increase in family conflicts, as the resources devoted to their personal lives are rather spent in maintaining their quality of work [40, 41]. It is likely that it is late in the process of burnout, only when personal and professional resources are depleted, that observable patient treatment will be adversely affected [4].

Nevertheless, a study in our review found an alarming effect of burnout on standardized mortality in Swiss ICUs [19]. The study investigated both physicians and nurses in these units (*N* = 1425), and the predictor model (where merely emotional exhaustion was significant) contributed with only 10% of the explained variance in the regression towards mortality. Other important variables such as patient characteristics and various work-related psychosocial factors were not included in the predictor model.

The strengths of this study are the aggregation of a large number of participants from the included studies and diversity of countries where the studies have been performed. Our search was limited to studies published since 2007, and among the 11 studies included in this review, two encompassed both physicians and nurses in the sample [19, 26]. By only including studies with observable measures for adverse patient treatment, we did not consider patient satisfaction, though it is an important indicator of quality in modern medicine. One limitation is that we did not include other burnout measures such as, e.g., OLBI and CBI. The time limits for the review could have been broader, since this literature is rather limited, and we may have missed some studies before 2007. The lack of studies with objective measures of quality of care might also be due to publication bias because negative outcome studies may not have been published.

Future research should investigate possible links between burnout and impaired quality of patient care. Studies of this kind can be a challenge to design and costly to perform. Some might even be questionable from an ethical point of view, for example designing a randomized controlled trial with an expectation that one of the groups will deliver less sufficient medical treatment. We also need more non-Western studies on this issue.

## Conclusion

In our review, only four, and in part a fifth, of 11 studies found a relationship between physician burnout measured by MBI and observed adverse patient outcomes.

More studies should be performed to further understand whether burnout among physicians leads to lowered patient care, and in particular to validate the specific level of burnout that impairs physician’ functioning with respect to observed adverse patient outcomes.

## Data Availability

Data sharing is not applicable to this article as no datasets were generated or analysed during the current study.

## Abbreviations

MBI: Maslach Burnout Inventory
DP: Depersonalization
EE: Emotional exhaustion
PA: Personal accomplishment (reduced)
OLBI: Oldenburger Burnout Inventory
CBI: Copenhagen Burnout Inventory
GP: General practitioner
ICU: Intensive care unit
